# Can late language learners acquire second language grammar? Evidence from linguistic to neuroscience methods

**DOI:** 10.3389/fpsyg.2024.1421072

**Published:** 2024-07-03

**Authors:** Yang Li

**Affiliations:** Psychology, Department of Social Sciences and Nursing, Solent University, Southampton, United Kingdom

**Keywords:** second language (L2) acquisition, psycholinguistic, neurolinguistic, late language learners, Universal Grammar (UG)

## Introduction

Although nowadays many individuals are learning another language, achieving native-like competence is still a challenge for many. Previous research suggests various factors influencing the final achievement of second language acquisition, including the similarity between L1 and L2 (e.g., White, 2003), the age of L2 acquisition (e.g., Singleton and Ryan, 2004), the quality and length of L2 input (e.g., Long, 1996), and so on. In 1967, Lenneberg proposed the critical period hypothesis, which states that the ability to learn language to a native-like proficiency is lost after a particular age. Previous research has demonstrated that grammar, compared with other domains of language, is more vulnerable to the critical period hypothesis (e.g., Yuan, 2010). This article aims to consolidate evidence from linguistic, behavioral, and neuroscience methods to provide a comprehensive summary of the topic.

## Linguistic evidence

One group of researchers (e.g., Lardiere, 2000; Prévost and White, 2000a) proposed that late L2 learners can acquire second language grammatical features, since interlanguage grammar is subject to the constraints of Universal Grammar in the functional domain. Therefore, L2 grammar can fully utilize functional categories.

Lardiere (1998a,b, 2000) conducted a series of studies examining an adult Chinese learner of English, Patty, who had been staying in America for many years. Although Patty's performance in supplying English morphology is low (e.g., 17% 3rd person singular agreement), Lardiere still argues that Patty had acquired the functional categories associated with verb inflection that don't exist in her native language. This argument is supported by three pieces of evidence. Firstly, Patty demonstrates the ability to accurately assign case to pronouns and indicate the function of finiteness, which suggests the presence of Tense in Patty's syntactic representation. In addition, Patty does not produce thematic verb-raising sentences, indicating her correct understanding that T in English has weak inflectional properties. The final piece of evidence is based on an assumption: the presence of a Complementizer Phrase (CP) implies the existence of all lower functional projections. Patty demonstrates the ability to produce various types of CP clauses. Lardiere concluded that Patty's syntactic feature development was complete and that she could acquire Tense Phrase and Agreement Phrase.

So, what caused Patty's poor performance when producing English inflectional morphology? Lardiere (2000) proposed that Patty's difficulty lies in the “mapping” of morphology to her previously acquired syntactic representations. It is within the mappings from morphology to Phonetic Form that are most likely to encounter “fossilization.” Furthermore, Lardiere (1998a) suggested that phonological transfer from Patty's first language may also contribute to the challenge of morphological spell-out since Mandarin does not permit final consonant clusters. Patty frequently deleted -t/-d in both inflected and monomorphemic forms.

Lardiere's explanation has laid the foundation for two later proposed hypotheses, the Missing Surface Inflection Hypothesis (Haznedar and Schwartz, 1997; Lardiere, 2000; Prévost and White, 2000a,b), and the Prosodic Transfer Hypothesis (Goad et al., 2003; Goad and White, 2004). These two hypotheses believe that late L2 learners' syntactic representations are intact. In terms of variation in inflection production by late L2 learners, the Missing Surface Inflection Hypothesis suggests it was due to the mapping problem while the Prosodic Transfer Hypothesis suggests it was due to the influence of L1 prosodic phonology.

Other researchers (e.g., Hawkins and Chan, 1997) state that late L2 learners' interlanguage representation is defective and therefore it is unlikely to achieve native-like competence level. For example, Hawkins and Chan (1997) proposed the Failed Functional Feature Hypothesis, arguing that adult learners are unable to acquire second language functional categories that are unavailable in their native language. In 1995, Chomsky introduced the Minimalist Program, which categorizes syntactic features into interpretable (with semantic meaning) and uninterpretable (without semantic meaning) features. Correspondingly, Tsimpli and Dimitrakopoulou (2007) make more precise claims about the representational deficit in L2 interlanguage grammar in the Interpretability Hypothesis. According to the Interpretability Hypothesis, uninterpretable features are subject to critical period constraints, while interpretable features, even those not presented in L1, remain available to late L2 learners.

In conclusion, the point of controversy for researchers in the fields of linguistics lies in whether late L2 learners can acquire features that don't exist in their native language.

## Behavioral evidence

The research discussed earlier relied on grammaticality judgment and oral production tasks. However, researchers argue that data from these two methods do not directly reflect individuals' language competence (Gass, 1994). To address this, researchers have employed the self-paced reading task to explore this question. In these studies, participants read both grammatical and ungrammatical sentences word by word. Detecting slower reaction times for critical words/phrases in ungrammatical sentences compared to their grammatical counterparts indicated participants' detection of syntactic violations and acquisition of relevant syntactic features. Given that the time to process the critical words/phrases is short, participants are less likely to apply metalinguistic knowledge (Carreira and Kagan, 2011).

Research using self-paced reading has yielded differing opinions on whether late L2 learners can acquire second language grammar, even when testing the same group with identical syntactic features. For instance, Wen et al. (2010) found that late advanced Chinese and Japanese English learners, whose native languages lack inflectional morphemes, could acquire English inflectional morphemes. However, Jiang (2004, 2007) found that advanced Chinese English learners failed to demonstrate the acquisition of English number agreement. This prompts the question of why Jiang (2004, 2007) and Wen et al. (2010) obtained conflicting results. According to Wen et al. (2010), they used simpler Noun Phrases (NPs) while Jiang's (2007) used more complex NPs. Additionally, Wen et al. (2010) questioned whether all participants in Jiang (2004, 2007) studies had reached an advanced level of English proficiency since Jiang used TOEFL scores to recruit participants and these scores might outdated. Table 1 summarizes previous research, showing diverse findings on whether late L2 learners can acquire L2 grammar.

**Table 1:** Summary of research using self-paced reading to study second language grammar acquisition by late L2 learners.

**References**	**Participants**	**L2 syntactic features**	**Main findings**
Jiang (2004)	Late Chinese learners of English and English native speakers	Number agreement, Pronoun agreement and Subcategorization Specification	Late Chinese learners of English do not exhibit sensitivity to number disagreement, but they do demonstrate sensitivity to other idiosyncrasies tested.
Jiang (2007)	Late Chinese learners of English and English native speakers	Number agreement and Verb subcategorization	Native English speakers were sensitive to errors involving plural-s and verb subcategorization. Chinese learners of English only showed sensitivity to verb subcategorization errors.
Wen et al. (2010)	Intermediate and advanced Chinese and Japanese English learners	Number agreement	Both advanced Chinese and Japanese English learners demonstrated sensitivity to English number disagreement.
Foote (2011)	Early and late English learners of Spanish; Spanish native speakers	Subject-verb agreement; Adjective gender agreement	Both early and late L2 learners of Spanish demonstrate sensitivity to subject-verb number agreement and non-adjective gender agreement.
Coughlin and Tremblay (2013)	Intermediate and advanced French learners of English and English native speakers	Number agreement	Advanced French learners of English and native English speakers both demonstrate sensitivity to number disagreement.
Mueller and Jiang (2013)	Advanced late L2 Korean speakers and Korean native speakers	Honorific Affix	Only Korean native speakers showed sensitivity to errors related to honorific affix.
Roberts and Liszka (2013)	Advanced late French, German English learners and English native speakers	Past simple and present perfect tense	French L2 learners show sensitivity to both past simple and present perfect mismatch conditions; German L2 learners showed no processing cost for either. English native speakers demonstrate sensitivity only to present perfect mismatch conditions.
Jegerski (2016)	Advanced and near native late L2 Spanish speakers; Native Spanish speakers	Subject-verb agreement	Both native and near-native L2 Spanish speakers demonstrate native-like online sensitivity to verbal number agreement.
Yao and Chen (2017)	Advanced and low proficiency late Chinese learners of English; English native speakers	Progressive, Past tense and 3rd person singular	Both high and advanced late Chinese English participants demonstrate sensitivity to the violation of the progressive. High-proficiency late Chinese English participants were sensitive to the violation of the past tense. They failed to demonstrate sensitivity to the violation of the third person singular in the self-paced reading task but successfully demonstrated sensitivity in the eye-tracking task.

## Neuroscience evidence

Accumulating electrophysiological studies have also been conducted to investigate whether late L2 learners can acquire second language grammar (e.g., Weber-Fox and Neville, 1996; Hahne and Friederici, 2001). Two Event-Related Potentials (ERP) components have been frequently used: P600 and Left Anterior Negativity (LAN). The P600 is a late positivity occurring between 500 and 1,000 ms with a posterior scalp distribution (e.g., Osterhout and Holcomb, 1992; Coulson et al., 1998). It reflects syntactic reanalysis processes followed by the detection of apparent ungrammaticality (Friederici, 1995; Münte et al., 1997). The LAN is a negative evoked component elicited between 300 and 600 ms after the presentation of a target word in a sentence. The LAN can be interpreted as a direct response to syntactic or morphosyntactic ungrammaticality (e.g., Osterhout and Holcomb, 1992; Münte et al., 1993), and this process is distinguished by a high level of automaticity (Gunter et al., 2000). The scalp topography of LANs has been varied across studies (Tanner and Van Hell, 2014).

The results obtained from ERP methods concerning whether late L2 learners can acquire L2 grammar are controversial, mirroring findings from linguistic and behavioral methods. Steinhauer et al. (2006) studied late French and Chinese learners of English processing syntactic word category violations. English native speakers exhibited a biphasic LAN/P600 response. High proficiency French and Chinese learners of English, similar to English native speakers, also exhibited a biphasic LAN/P600 response. Therefore, Steinhauer et al. (2006) concluded that late L2 learners can exhibit native-like ERP patterns provided they achieve proficiency in their second language. However, in other studies, Sabourin and Stowe (2008) found that late learners can only exhibit native-like brain patterns for second language grammar features similar to their first language. For second language grammar features that operate differently from their first language, even late second language learners who have achieved high proficiency still struggle to generate native-like brain patterns. Similar findings have been obtained in other studies (e.g., Ojima et al., 2005; Chen et al., 2007). Previous research also found late L2 learners receive implicit input during language learning and achieve L2 high proficiency, they can achieve native-like ERP patterns for L2 grammar errors (Short, 2007; Bowden et al., 2013).

Studies utilizing fMRI have highlighted the significance of whether a learner acquires their second language early or late in life in determining how their brain processes second language grammar (Wartenburger et al., 2003; Rüschemeyer et al., 2005; Liu and Cao, 2016; Oh et al., 2019). Particularly noteworthy, Wartenburger et al. (2003) found that only early L2 learners showed identical brain activity as native speakers while late L2 learners, independent of their second language proficiency, demonstrated increased activity in the bilateral inferior frontal gyrus. These results have been confirmed by later research, which suggested there is more activity in the prefrontal cortex when late L2 learners process L2 grammar, compared with native speakers or early L2 learners (Luke et al., 2002; Oh et al., 2019). These results are consistent with the idea that there is a “critical period” for second language grammar and provide empirical research support for the sensorimotor/emergentist model (Hernandez and Li, 2007; Hernandez et al., 2007) and the declarative/procedural model (Ullman, 2004, 2016) as both models suggest that different brain areas are involved in processing early acquired and late acquired grammar.

However, differing opinions exist in the field. For instance, Abutalebi (2008) argued that neural representation for second language acquisition is identical to that of first language acquisition, even for late L2 learners. As to why some of the research found that late L2 learners use different neurocognitive structures from native speakers, Abutalebi (2008) argued that it is because participants in these studies have low second language proficiency.

## Discussion

The fields of linguistics, behavioral studies, and neuroimaging have yielded conflicting findings on the acquisition of second language grammar by late learners. However, findings in the fields of neuroscience and traditional linguistic methods agree that even late L2 learners can acquire L2 syntactic features that exist or operate similarly to those in their native language (e.g., Tsimpli and Dimitrakopoulou, 2007; Sabourin and Stowe, 2008). Therefore, more research should be conducted on whether late L2 learners can acquire L2 syntactic features that do not operate in their first language and do not contain any semantic meaning.

Supporters of the accessibility of second language grammar to late learners often challenge proponents of the critical period, arguing that their participants' proficiency in the second language may not be sufficiently high to demonstrate comparable performance to native speakers (e.g., Abutalebi, 2008; Wen et al., 2010). This is difficult to assess since most of the previous research lacks an independent proficiency test. The absence of independent proficiency research is a common issue in psycholinguistic studies. Lemhöfer and Broersma (2012) found that only five out of 18 studies published in top experimental psychology journals between 2009 and 2011 used independent proficiency tests. Previous research primarily relied on language background questionnaires and self-assessment measures, which researchers questioned as sufficient for objectively measuring participants' language proficiency (e.g., Delgado et al., 1999). Therefore, future research should incorporate independent proficiency tests or even a test assessing their knowledge of specific grammar features under investigation to validate participants' language proficiency.

Apart from that, another factor that needs attention is the difficulty of the research materials. Even when testing the same syntactic features, the difficulty of research materials varies across studies (e.g., Jiang, 2007; Wen et al., 2010), leading to contrasting findings. McDonald (2006) suggests that late second language learners perform worse than native speakers in tasks involving working memory, decoding, and speed, which are correlated with the accuracy of L2 grammaticality judgment tasks. Furthermore, the ERP component P600, frequently used to index syntactic processing, is also influenced by processing difficulty (e.g., Brouwer et al., 2012) and working memory (e.g., Alatorre-Cruz et al., 2018). Therefore, research adopting difficult materials may risk attributing their conclusions to the processing difficulties rather than the critical period.

## Conclusion

In conclusion, the question of whether late L2 learners can acquire second language grammatical features remains crucial. With the increasing available research methods, future research should consider employing combined methods. For example, integrating grammaticality judgment tasks with neuroimaging studies and correlating results from different research methods could provide deeper insights into this issue.

## Author contributions

YL: Writing – original draft, Writing – review & editing.
